# MicroRNA-466 inhibits tumor growth and bone metastasis in prostate cancer by direct regulation of osteogenic transcription factor RUNX2

**DOI:** 10.1038/cddis.2017.15

**Published:** 2017-01-26

**Authors:** Melissa Colden, Altaf A Dar, Sharanjot Saini, Priya V Dahiya, Varahram Shahryari, Soichiro Yamamura, Yuichiro Tanaka, Gary Stein, Rajvir Dahiya, Shahana Majid

**Affiliations:** 1 Department of Urology, VA Medical Center and UCSF, San Francisco, CA, USA; 2 CPMC Research Institute, San Francisco, CA, USA; 3 Department of Biochemistry and Surgery, University of Vermont College of Medicine, 148 Beaumont Avenue, Burlington, VT, USA

## Abstract

**MicroRNAs (miRNAs) have emerged as key players in cancer progression and metastatic initiation yet their importance in regulating prostate cancer (PCa) metastasis to bone has begun to be appreciated. We employed multimodal strategy based on in-house PCa clinical samples, publicly available TCGA cohorts, a panel of cell lines, *in silico* analyses, and a series of *in vitro* and *in vivo* assays to investigate the role of miR-466 in PCa. Expression analyses revealed that miR-466 is under-expressed in PCa compared to normal tissues. Reconstitution of miR-466 in metastatic PCa cell lines impaired their oncogenic functions such as cell proliferation, migration/invasion and induced cell cycle arrest, and apoptosis compared to control miRNA. Conversely, attenuation of miR-466 in normal prostate cells induced tumorigenic characteristics. miR-466 suppressed PCa growth and metastasis through direct targeting of bone-related transcription factor RUNX2. Overexpression of miR-466 caused a marked downregulation of integrated network of RUNX2 target genes such as osteopontin, osteocalcin, ANGPTs, MMP11 including Fyn, pAkt, FAK and vimentin that are known to be involved in migration, invasion, angiogenesis, EMT and metastasis. Xenograft models indicate that miR-466 inhibits primary orthotopic tumor growth and spontaneous metastasis to bone. Receiver operating curve and Kaplan–Meier analyses show that miR-466 expression can discriminate between malignant and normal prostate tissues; and can predict biochemical relapse. In conclusion, our data strongly suggests miR-466-mediated attenuation of RUNX2 as a novel therapeutic approach to regulate PCa growth, particularly metastasis to bone. This study is the first report documenting the anti-bone metastatic role and clinical significance of miR-466 in prostate cancer.**

Prostate cancer is the second most leading cause of cancer related deaths among American men.^1^ According to recent data, it is estimated that 220, 800 newly diagnosed prostate cancer cases and 27 540 deaths will occur in 2015.^1^ The 5 year relative survival rate of early stage prostate cancer is >99% while that of advanced metastatic disease is only 28%.^1^ Metastases often occur with no prior indication of tumor invasiveness.^2^ A major challenge for treatment of advanced metastatic disease is the lack of understanding of the molecular mechanisms underlying the propensity of prostate cancer to metastasize to other organs, particularly the bone. A number of transcription factors have been identified that play key roles in promoting oncogenesis, tumor growth, metastasis and tissue destruction.

Runt-domain containing protein RUNX2 (also called Osf2/Cbfa1, AML-3 or Pebp2*α*A) is a member of the RUNX family of genes. It is a lineage-specific transcription factor with crucial roles in both bone biology and carcinogenesis.^3, 4, 5^ High levels of RUNX2 in metastatic prostate cancer emphasize the significance of this master skeletal transcription factor in potentiating tumor cell progression and metastatic bone disease.^6, 7, 8^ The oncogenic potential of RUNX2 was first documented T-cell lymphoma.^9, 10^ Since then, a number of independent studies have reported involvement of RUNX2 at early and late stages of tumor progression in various cancers.^6, 7, 11, 12, 13, 14, 15^ RUNX2 was found to be up-regulated and a predictor of metastasis in prostate cancer.^16^ Mechanistic studies indicated that RUNX2 accelerated prostate cancer aggressiveness through promotion of cadherin switching, invasion toward collagen I and Akt activation.^16^ In addition, attenuation of RUNX2 expression in a PC3 subline led to increased adhesion to fibronectin.^7^ Various reports have convincingly shown that the severity of prostate and mammary cancer cell osteolysis is correlated with levels of RUNX2 expression following injection of neoplastic cells in the medullary cavity of the tibia.^7, 8, 14^ These findings suggest that RUNX2 can imbue neoplastic cells with the capacity to breakdown the architecture of surrounding tissues, potentially releasing tumor supporting factors indicating that RUNX2 may facilitate a bone-mimetic program that helps cells adapt and thrive in this foreign environment. RUNX2 also directly induced genes associated with angiogenesis, invasiveness, metastasis and stimulated epithelial-to-mesenchymal transition of primary tumors.^15, 16, 17^ Taken together these studies provide convincing evidence for RUNX2 as a potentially important factor in prostate cancer development and metastasis. Targeting RUNX2 in prostate cancer could interrupt an integrated network of gene expression required to maintain tumor growth and bone metastasis. Here we report use of miR-466 as a potentially novel avenue to regulate RUNX2 and its downstream genes, thereby inhibiting prostate cancer growth and bone metastasis.

MicroRNAs (miRNA) are small (20–25 nucleotides) evolutionarily conserved non-coding RNAs that negatively regulate transcript levels through sequence-dependent recognition mechanisms.^18^ Over the past several years it has become clear that alteration in the expression of miRNA genes contribute to the pathogenesis of many human malignancies. Carcinogenesis involves multiple genetic and epigenetic events, yet the organizing principles underlying their choreography are poorly understood. miRNA deregulation is an important component of this landscape given the oncogenic and tumor-suppressive functions of miRNAs.^18^ Since malignant cells have dysregulated expression of miRNAs, which in turn control or are controlled by the dysregulation of multiple protein-coding oncogenes or tumor suppressor genes, these miRNAs may be important for the development of miRNA-based therapies.^18^ In addition miRNAs have great potential as diagnostic and prognostic biomarkers. Owing to their tissue specificity, miRNAs have become useful tools for defining the origin of tumors in poorly differentiated cancers.^19^ With the advent of miRNA expression profiles, significant efforts have been made to correlate miRNA expression with tumor prognosis.^20, 21, 22, 23^ These reports even suggest that expression profiling of miRNAs may be a more accurate method of classifying cancer subtypes than using the expression profiles of protein-coding genes.^22, 24^ Various studies have established miRNA expression patterns as potential biomarkers for diagnosis, prognosis, personalized therapy, disease management and clinical outcome in various cancers.^25, 26, 27, 28, 29, 30^ To date, 28 645 miRNAs have been identified (http://www.mirbase.org) although their role in disease pathogenesis is not well documented.

Here for the first time we show that miR-466 inhibits prostate cancer bone metastasis. miR-466 is significantly under-expressed in a panel of prostate cancer cell lines and clinical tissues compared to normal. Restoration of miR-466 expression in highly metastatic PC3 and Du145 prostate cancer cell lines impaired proliferation, migration, invasion and induced cell cycle arrest and apoptosis *in vitro*. Importantly, miR-466 has a profound inhibitory effect on prostate cancer growth and bone metastasis *in vivo*. We further demonstrated that miR-466 partially exerted these effects by directly targeting runt-related RUNX2, a master osteogenic transcription factor and attenuated its downstream target genes that are known to mediate prostate cancer bone metastasis. In addition, our data indicates that miR-466 is potentially a clinically significant prostate cancer biomarker.

## Results

### miR-466 expression is significantly downregulated in prostate cancer

**Figure 1:**
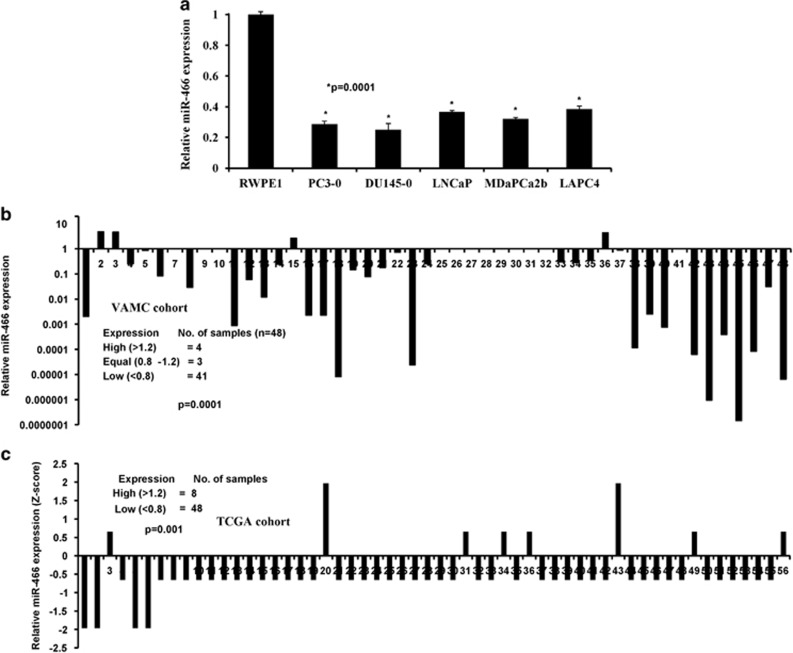
MiR-466 expression is downregulated in prostate cancer (**a**) Expression of miR-466 was analyzed by qRT-PCR in a panel of prostate cancer lines compared to a non-malignant cell line (RWPE1). (**b**) Expression of miR-466 in a patient cohort (*n*=96; 48 pairs) of matched LCM tissue samples from in-house VAMC cohort. (**c**) Confirmation of miR-466 expression in an independent validation cohort of prostate adenocarcinoma (*n*=56) publicly available at the TCGA data base

In a previous study,^31^ we performed preliminary screening to identify differentially expressed miRNAs in prostate cancer cell lines compared to a non-malignant cell line. A set of miRNAs, miR-466, -205, -203, -23b and -34b were found to be significantly downregulated in prostate cancer cells compared to a non-malignant cell line. We validated miR-466 data by miRNA-quantitative real-time PCR (miR qRT-PCR) analysis. The results confirmed that miR-466 was significantly downregulated in a panel of prostate cancer cell lines compared to normal RWPE1 cells (Figure 1a). Further, miR-466 expression was analyzed in an experimental cohort of laser-captured micro-dissected (LCM) matched patient samples from the San Francisco VA Medical Center (VAMC cohort) and attenuation of miR-466 expression was confirmed (Figure 1b). The expression of miR-466 was downregulated in 85% of PCa tissue samples compared to matched normal tissues (*P*<0.0001; Figure 1b). Suppression of miR-466 in the experimental cohort was validated by analyzing an independent cohort of prostate adenocarcinoma available publicly at The Cancer Genome Atlas (TCGA) data base (*P*<0.001; Figure 1c). These results indicate a putative tumor suppressor role for miR-466 in prostate cancer. We reason that tumor suppressor miRNAs would be expressed at low levels in tumors but more highly expressed in normal tissues. MiR-466 fulfills this criterion, exhibiting high expression in normal prostate tissues and a non-malignant cell line and low or silenced expression in prostate cancer samples and cell lines.

### miR-466 overexpression suppresses cell proliferation, migration, invasion, reverses epithelial-to-mesenchymal-transition (EMT) and induces cell cycle arrest and apoptosis in prostate cancer

**Figure 2:**
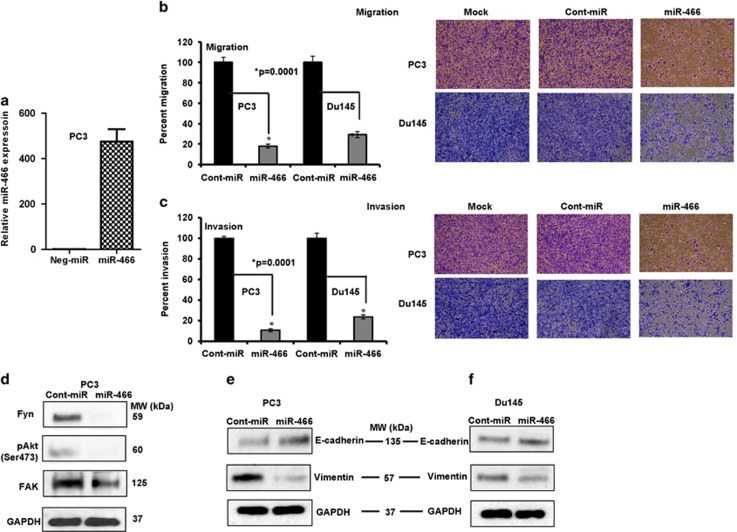
Functional significance of miR-466 in prostate cancer. (**a**) Expression of miR-466 after transient transfection of miR-466 mimic in PC3 cells compared to a scrambled negative control miRNA (Neg-miR). (**b** and **c**) PC3 and Du145 cells expressing miR-466 were subjected to transwell assay to evaluate chemotactic migration (**b**) and invasion (**c**). Assays were performed after 18 h of transient transfection of miR-466 in PC3 and Du145 cells. Data are represented as the mean±s.d. Images are those of mock, neg-miR and miR-466 transfected groups. (**d**–**f**) Immunoblot assay of endogenous levels of proteins involved in proliferation, migration, invasion and EMT in PC3 and Du145 cells transfected with miR-466 or neg-miR. GAPDH was used as an internal control

To ascertain the functional significance of miR-466 overexpression in prostate cancer cells, we performed migration, invasion, proliferation, cell cycle and apoptosis assays. Transient transfection of miR-466 mimic (50 nM for 72 h; Figure 2a) caused a significant decrease in migration (Figure 2b) and invasion (Figure 2c) of PC3 and Du145 prostate cancer cells. We also examined the effect of overexpression of miR-466 on markers involved in migration, invasion and EMT. Ectopic expression of miR-466 significantly decreased Fyn, Fak and pAkt protein levels that are implicated in migration and invasion of human prostate cancer (Figures 2d–f). In addition, a decrease in Vimentin (mesenchymal marker) and an increase in E-cadherin (epithelial marker) was observed in miR-466 transfected cells compared to cont-miR transfection (Figures 2e and f). EMT is involved in epithelial-derived tumors causing them to become invasive and metastatic. These results show that miR-466 suppressed EMT markers along with other migratory/invasive genes attesting to the metastatic suppressor role of miR-466 *in vitro*.

**Figure 3:**
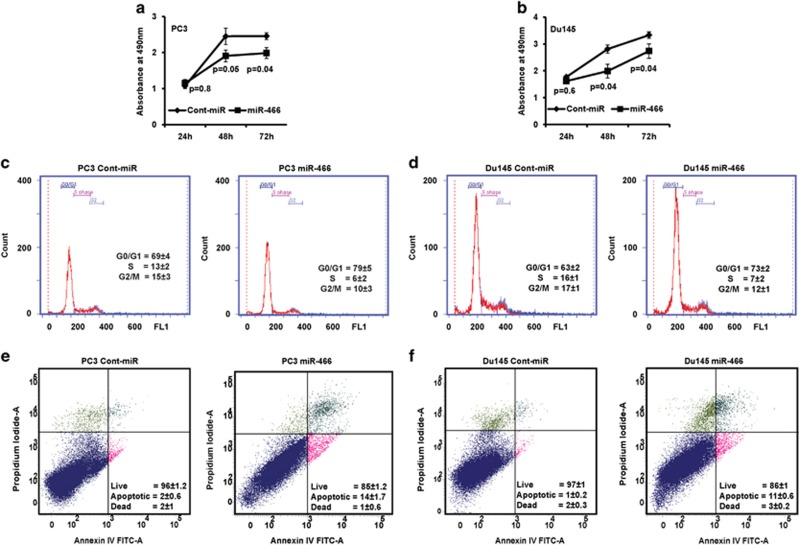
Reconstitution of miR-466 inhibits cell proliferation and induces cell cycle arrest and apoptosis in prostate cancer. (**a** and **b**) Proliferation of PC3 and Du145 cells ectopically expressing miR-466 or neg-miR was determined by MTS assay. (**c** and **d**) FACS cell cycle and (**e** and **f**) apoptosis analyses of PC3 and Du145 cells expressing miR-466 compared to negative control miRNA. Values are mean of three independent experiments±S.D. and the quadrants are representatives of the experiment

Unregulated cell proliferation and survival are common features of cancer cells that favor metastatic dissemination. Reconstitution of miR-466 in metastatic PC3 and Du145 prostate cancer cells inhibited cell proliferation (Figures 3a and b), induced G0/G1 cell cycle arrest (Figures 3c and d) and apoptosis (Figures 3e and f) compared to control miRNA. These results confirm the tumor suppressor phenotypic effects of miR-466 overexpression in human prostate cancer cells.

### miR-466 inhibits primary tumor growth and bone metastasis *in vivo*

**Figure 4:**
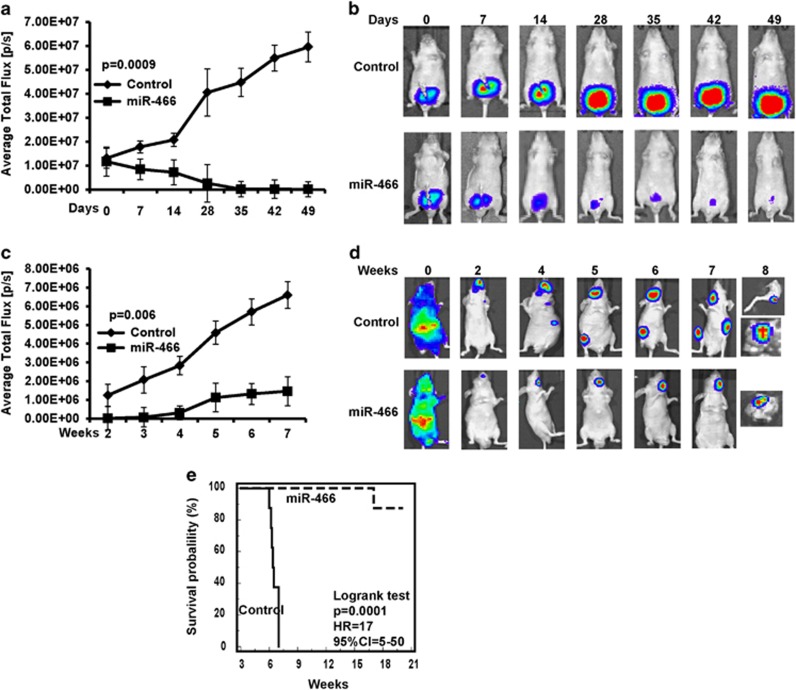
MiR-466 inhibits primary tumor growth and bone metastasis *in vivo*. (**a** and **b**) Primary tumor growth in an *in vivo* intraprostatic xenograft model was determined by quantifying the bioluminescent signal after orthotopically transplanting bone metastatic PC3 cells stably expressing miR-466. (**c** and **d**) miR-466 significantly inhibited spontaneous metastases to bone indicated by the bioimaging of mice after intracardiac implantation of constitutively miR-466 expressing PC3 cells. (**b** and **d**) Representative images of mice from each group of *in vivo* models. (**d**8) Bioluminescent signal in harvested leg and skull of control and miR-466 group. (**e**) Kaplan–Meier survival curve based on intracardiac spontaneous metastatic model. In **a** and **b** data are represented as mean±S.E.M. of each group

To recapitulate the *in vitro* findings and to examine the role of miR-466 in tumorigenesis *in vivo*, bioluminescent PC3M-Luc-C6 cells (1 × 10^6^ cells) constitutively expressing cont-miR or miR-466 were orthotopically implanted into the posterior prostatic lobe of athymic nude mice (Figure 4a). Mice were bioimaged immediately to check equal distribution of cells as indicated by the luciferase signal intensity at day 0 in both groups. Regular monitoring of mice with the IVIS imaging system was performed on a weekly basis. miR-466 significantly reduced the primary tumor size as determined by bioluminescence imaging over the course of the experiment (Figures 4a and b) compared to control. Mice in both groups survived until the end of the experiment. In view of the robust inhibitory effect of miR-466 on migration and invasion of prostate cancer cells *in vitro,* we reasoned that it may also inhibit metastasis *in vivo*. Indeed, miR-466 suppressed spontaneous metastasis to bone 7 weeks after intracardiac injection compared to controls (Figures 4c and d). Control cells grew aggressively and metastatic lesions were observed in the knee, jaw and ribs whereas miR-466 drastically inhibited bone metastasis. Mice were monitored for survival and all eight control mice either died or had to be killed because of tumor burden by the 7th week. In the miR-466 mouse group 7 of 8 mice survived until the 20th week when the experiment was terminated. We performed Kaplan–Meier survival analysis and the results indicated that low-miR-466 expression strongly (*P*<0.0001) correlates with poor overall survival compared to the high miR-466 expression group (HR=17; 95% CI=5–50; Figure 4e). These results confirm that miR-466 functions as a significant tumor and bone metastasis suppressor gene in prostate cancer.

### miR-466 directly represses osteogenic transcription factor RUNX2

**Figure 5:**
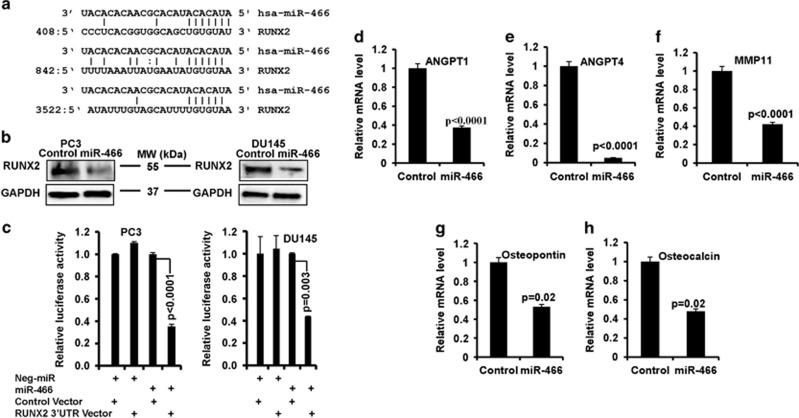
MiR-466 directly regulates osteogenic transcription factor RUNX2 and attenuates RUNX2 target genes related to tumor growth and bone metastasis. (**a**) Schematic representation of miR-466 complimentary binding sites in the RUNX2 3′UTR by *in silico* computational algorithms (microrna.org and mirdb.org). (**b**) Endogenous RUNX2 protein was detected in miR-466 and neg-miR (control) tranfected PC3 and Du145 cells by immunoblot analysis. GAPDH was used as a loading control. (**c**) Luciferase-RUNX2 reporter assays. miR-466 and neg-miR were transfected into PC3 and Du145 cells co-transfected with RUNX2 3′UTR or control non-specific plasmid construct. Firefly luciferase activity was normalized to co-transfected Renilla luciferase and presented as relative luciferase activity. Data are represented as mean±S.D. (**d**–**h**) Expression of RUNX2 target genes including ANGPT1, ANGPT4, MMP11, osteopontin (SPP1) and osteocalcin (OC) was analyzed in miR-466 and neg-miR (control) transfected cells by qRT-PCR. Data are represented as mean±S.D.

We next sought to determine the underlying molecular mechanism of miR-466 mediated inhibition of tumor growth and bone metastasis. Thus, we utilized *in silico* computational algorithms to identify miR-466 target genes involved in these processes. Two different miRNA databases (microrna.org; mirdb.org) identified three complimentary miR-466 binding sites in the 3′UTR of RUNX2 (Figure 5a). Ectopic expression of miR-466 significantly attenuated RUNX2 protein levels, suggesting a functional role in controlling protein translation (Figure 5b). Luciferase reporter assays validated that miR-466 directly targets the wild-type 3′UTR of RUNX2 as co-transfection of the miR-466 along with wild-type RUNX2 3′UTR significantly repressed relative luciferase activity (Figure 5c) in PC3 and Du145 cells. No effect was observed with cont-miR or miR-466 cells transfected with a non-specific 3′UTR control vector (Figure 5c).

### miR-466 suppresses multiple RUNX2 target genes related to tumor growth and bone metastasis

Next we determined the effect of miR-466 mediated RUNX2 suppression on downstream pathway genes. As a consequence of inhibited RNUX2 expression, several RUNX2 target genes associated with angiogenesis, invasiveness and metastasis including ANGPT1, ANGPT4, Osteopontin (Spp1), Osteocalcin (OC) and MMP11, were downregulated by miR-466 compared with control cells (Figures 5d–h). These results further support our hypothesis that the effect of miR-466 in prostate tumorigenesis and metastasis is mediated through inhibition of RUNX2 and its metastasis-promoting downstream target genes.

### Knockdown of endogenous miR-466 in non-malignant RWPE1 cells induces pro-cancerous attributes

**Figure 6:**
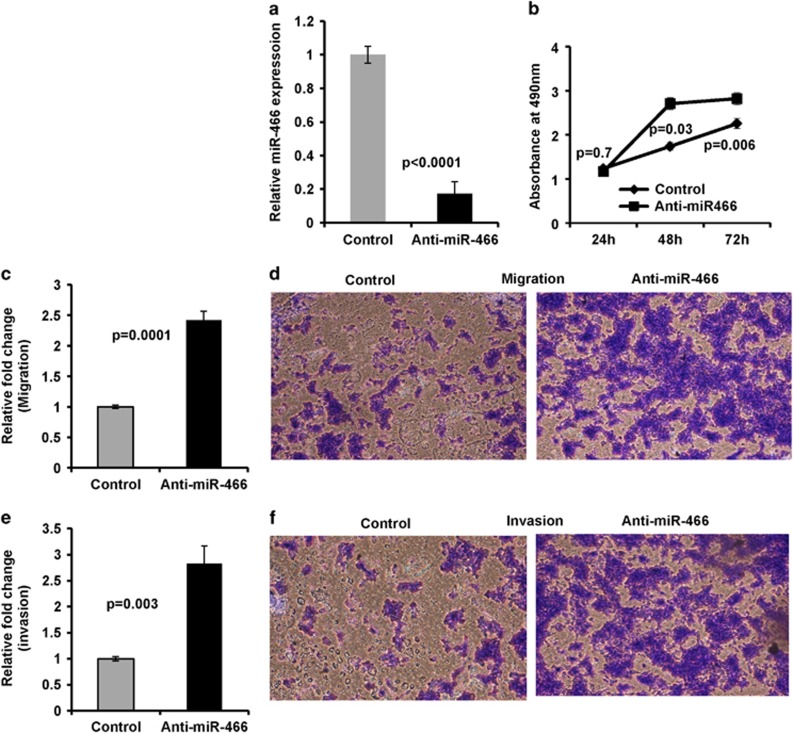
Knockdown of endogenous miR-466 in non-malignant RWPE1 cells induces pro-cancerous attributes. (**a**) Expression of miR-466 72 h post transfection of miR-466 inhibitor (anti-miR-466) or anti-control inhibitor (control) in RWPE1 cells. (**b**) Proliferation of RWPE1 transfected with anti-miR-466 or control analyzed by MTS assay. (**c**–**f**) Suppression of miR-466 in RWPE1 cells induces tumorigenic attributes in these non-malignant cells as indicated by chemotactic transwell migration (**c** and **d**) and invasion (**e** and **f**) assays. Data is represented as mean±S.D. Images are representatives from each group

To assess the biological significance of miR-466 in prostate cancer; we performed parallel experiments using non-cancerous RWPE1 cells. miR-466 was knocked down in RWPE1 cells by transient transfection of anti-miR-466 (miR-466 inhibitor; 50 nM) along with a negative-anti-miRNA-control (control; Figure 6a). RWPE1 cells transfected with anti-miR-466 showed a considerable increase in cell proliferation (Figure 6b), migration and invasion (Figures 6c–f) compared to controls. These data provide evidence that suppression of miR-466 caused RWPE1 cells to have cancer-like characteristics further attesting to its tumor suppressor role in prostate cancer.

### Clinical significance of miR-466 in prostate cancer

**Figure 7:**
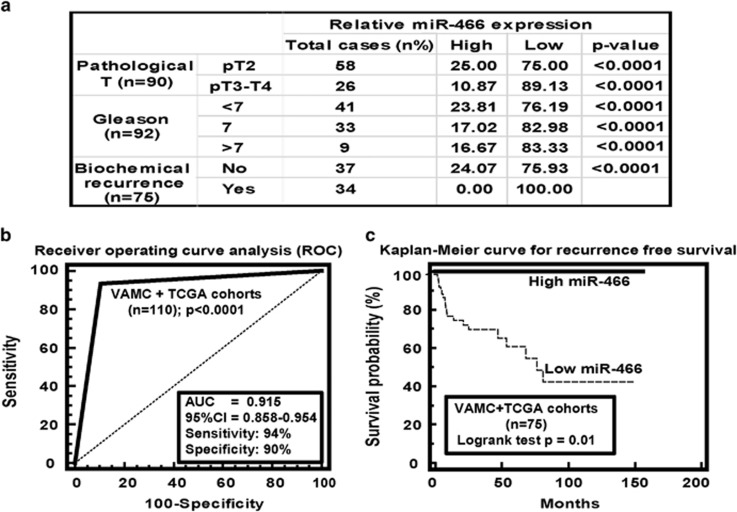
MiR-466 is a potential novel biomarker for prostate cancer. (**a**) *χ*^2^ test showing correlation of clinicopathological characteristics with miR-466 expression. (**b**) ROC curve analysis showing performance of miR-466 expression to discriminate between malignant and non-malignant prostate tissue samples. (**c**) Kaplan–Meier analysis for recurrence-free survival based on miR-466 expression. Note: different sample numbers in statistical analysis is because not all clinical information was available for all the samples

Statistical analyses were performed by combining the in-house VAMC cohort and prostate adenocarcinoma sample cohorts available at TCGA data portal. Clinical demographics of the study cohort are summarized in Supplementary Table 1. VAMC samples were dichotomized based on the relative expression of miR-466 and grouped into low (<1-fold relative to matched normal) and high (>1-fold) miR-466 expression groups; whereas TCGA samples were grouped as low (<mean expression) and high (>mean expression) groups. Correlation of miR-466 expression was assessed with clinic-pathological variables such as Gleason grade, pathological stage (pT) and biochemical recurrence (Figure 7a). The time of recurrence was defined as the first postoperative PSA value >0.1 ng/ml after surgery. Thirty-four patients experienced a biochemical relapse according to this criterion. A significant correlation was observed between miR-466 expression with clinical variables. In higher pathological grade samples (pT3-T4), decreased miR-466 expression was observed in 89% cases compared to 11% of cases that had high miR-466 expression (*P*<0.0001). In lower pathological stage (pT2) cases, decreased expression of miR-466 was observed in 75% of cases while 25% of cases showed high miR-466 expression (*P*<0.0001; Figure 7a). To analyze the correlation with Gleason score, we divided the samples into low <7, medium 7 and high >7 Gleason score groups. In the low group (<7), lower expression of miR-466 was found in 76% cases, whereas 24% cases had high miR-466 expression (*P*<0.0001). In the medium group (7), 83% of cases had low-miR-466 expression compared to 17% of cases with high miR-466 expression (*P*<0.0001). In the high group (>7), 83% of cases had low-miR-466 expression compared to 17% of cases with high miR-466 expression (*P*<0.0001; Figure 7a). In the PSA recurrence patients, 100% of cases had decreased miR-466 expression (*P*<0.0001; Figure 7a). These results reveal that number of cases with low-miR-466 expression increases from low Gleason grade, low pathological stage to high Gleason grade and high pathological stage. Interestingly, all PSA recurrence patients had low-miR-466 expression suggesting that miR-466 has clinical significance in prostate cancer.

Receiver operating curve (ROC) analyses were performed to evaluate the ability of miR-466 expression to discriminate between normal and tumor tissues. An area under the ROC curve (AUC) of 0.915 (*P*<0.0001; 95% CI=0.858–0.954; Figure 7b) was obtained suggesting that miR-466 expression can discriminate between malignant and non-malignant tissues and can be used as a diagnostic marker for PCa. As 100% of patients with biochemical relapse showed low-miR-466 expression, we performed Kaplan–Meier analysis to determine the ability of miR-466 to predict patient recurrence-free survival. Indeed, high miR-466 expression was predictive of better recurrence-free patient survival (*P*=0.02) compared to low-miR-466 expression (Figure 7c). Collectively, these results suggest that miR-466 has potential to be a diagnostic and prognostic marker for predicting prostate cancer biochemical recurrence though addition of more samples may strengthen these results.

## Discussion

Bone metastases are a major problem in the evolution of prostate cancer. Considerable effort has been devoted to map the requirements of bone lesions in prostate tumors.^32, 33^ Experimental evidence has demonstrated that RUNX2, a transcription factor essential for osteogenesis, is a key regulator of bone metastasis that becomes highly activated in prostate cancer cells that metastasize to bone.^34, 35^ RUNX2 has been found to be associated with the osteomimetic properties of bone metastatic prostate cancer cells via transcription of genes implicated in osteoblastic lesions including osteocalcin, osteopontin, VEGF and matrix metalloproteins (MMPs) and so on.^34, 35^ Thus, therapeutic targeting of RUNX2 may interrupt an integrated network of gene expression that trigger bone metastases and thus deregulate cell survival and migration pathways of invading prostate cancer cells. However, at present transcription factors such as RUNX2 are not considered to be drug targets.^33^ Here, we show that miR-466-mediated attenuation of RUNX2 may be a novel therapeutic approach to regulate tumor growth and bone metastasis in prostate cancer. Indeed, reconstitution of miR-466 in metastatic prostate cancer cells significantly attenuated RUNX2 protein levels, suggesting a functional role of miR-466 in controlling RUNX2 protein translation.

**Figure 8:**
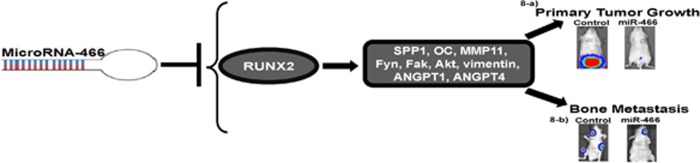
Schematic representation of the role of miR-466-mediated regulation of RUNX2 in prostate cancer. Reconstitution of miR-466 in prostate cancer leads to suppression of RUNX2 and its direct downstream target genes involved in tumor growth and bone metastasis resulting in inhibition of primary tumor growth (8-a) and bone metastasis (8-b)

miRNAs have emerged as key players in cancer progression and metastatic initiation yet the importance of miRNAs in regulating prostate cancer bone metastasis has just begun to be appreciated.^36^ In this study, we have identified miR-466 as a suppressor of prostate cancer growth and metastasis through direct targeting of the bone-related transcription factor RUNX2, which is difficult to target by conventional pharmacologic approaches.^33, 37^ Our *in vivo* xenograft studies provide evidence that miR-466 can inhibit primary orthotopic tumor growth and spontaneous metastasis to bone. Expression analyses based on our in-house VAMC patient cohort or the publicly available TCGA data cohort revealed that miR-466 is under-expressed in prostate cancer compared to normal tissues. Conversely RUNX2 has been detected in human prostate cancer tissues but not in normal prostate.^6, 34^ In this study, we have identified a tumor suppressor role for miR-466 in prostate cancer. Reconstitution of miR-466 in PC3 and Du145 metastatic prostate cancer cell lines inhibited *in vitro* oncogenic properties such as cell proliferation, migration and invasion compared to controls along with induction of G0/G1 cell cycle arrest and apoptosis in both cancer cell lines. MiRNAs control a wide range of biological functions and may act as tumor suppressors or oncogenes.^22^ Alteration of their expression plays a critical role in tumorigenesis and cancer progression.^21, 38^ Our study corroborates other reports that show miRNAs are linked to the biologic activities of RUNX2 in different cancer types.^37^ Recent studies have demonstrated the potential of miRNAs in the intervention of breast cancer progression, metastatic bone disease and other bone pathologies.^37, 39^ Interestingly, our study shows that the role of miR-466 in the control of prostate cancer progression and metastasis may be partially attributed to decreased cancer cell proliferation, cell cycle arrest and induced apoptosis, which features the beneficial effects of the miR-466 in regulating prostate cancer progression. However, our study highlights a direct role for miR-466 in reducing the bone metastatic potential of prostate cancer cells. This is evidenced by the fact that reconstitution of miR-466 caused a marked downregulation of RUNX2 target genes such as osteopontin, osteocalcin, ANGPT1, ANGPT4, MMP11 including Fyn, pAkt, FAK and vimentin that are involved in migration, invasion, angiogenesis, EMT and metastasis.^15, 16, 17, 34, 35^ Thus, miR-466 orchestrates the functional activities of RUNX2 by its convergent action on RUNX2 and its downstream target gene network to control prostate cancer bone metastasis. Furthermore, our study also defines the biological relevance of miR-466 in prostate cancer as indicated by induction of pro-cancerous characteristics in non-malignant RWPE1 cells after inhibition of endogenous expression of miR-466. Thus, our data strongly suggests a miR-466 mediated attenuation of RUNX2 may be a novel approach to prevent bone metastatic disease in prostate cancer (Figure 8).

miRNAs have great potential as diagnostic and prognostic biomarkers.^20, 21, 22, 23, 24, 25, 26, 27, 28, 29, 30^ Owing to their tissue specificity, miRNAs have become useful tools for defining the origin of tumors in poorly differentiated cancers^19^ and these reports even suggest that the expression profiling of miRNAs may be a more accurate method of classifying cancer subtype than using the expression profiles of protein-coding genes.^22, 24^ In this study, we found that miR-466 expression can significantly (*P*<0.0001) distinguish malignant from normal tissues indicating the powerful diagnostic potential of miR-466 in prostate cancer. In addition, miR-466 expression was predictive of recurrence-free survival (*P*<0.02) such that patients with higher miR-466 expression levels had better recurrence-free survival. Correlation analyses showed that low-miR-466 expression positively correlated with high pathological T (*P*=0.0001), Gleason grade (*P*<0.001) and PSA failure (*P*<0.0001). Therefore, our data reveals the clinical significance of miR-466 in prostate cancer, although it would benefit from a larger sample cohort.

In conclusion, for the first time we provide evidence that: (i) miR-466 is an under-expressed metastasis suppressor miRNA in PCa; (ii) miR-466 is biologically relevant and has biomarker potential in PCa; (iii) miR-466 directly regulates RUNX2, a key regulator of bone metastasis; (iv) miR-466 overexpression interrupts RUNX2 integrated network of genes required to maintain PCa growth and bone metastasis. RUNX2, a key transcription factor, is of pivotal significance in the pathogenesis and progression of PCa bone metastases. Therefore, restoration of miR-466 to regulate an integrated network of RUNX2 and its target genes may represent a promising approach to suppress prostate cancer bone metastases.

## Materials and Methods

### Cell culture, plasmids and probes/primers

Human prostate cancer cell lines PC3, Du145, LNCaP, MDaPCa2b and a non-malignant cell line (RWPE1) were obtained from the American Type Culture Collection (ATCC; Manassas, VA, USA) and grown according to ATCC protocol. These human-derived cell lines were authenticated by DNA short-tandem repeat analysis by ATCC. The experiments with cell lines were performed within 6 months of their procurement/resuscitation. Plasmids pEZX-MT01 miRNA 3′UTR target expression clones for RUNX2 and miRNA Target clone control vector for pEZX-MT01 were purchased from GeneCopoeia (Rockville, MD, USA). TaqMan probes including mimics, inhibitors and negative controls for hsa-miR-466 (miR-466) were purchased from Applied Biosystems (Foster City, CA, USA).

### Tissue samples, LCM, qRT-PCR and validation of expression in an independent TCGA data cohort

Tissue samples from radical prostectomy (*n*=96; 48 pairs) were obtained from the Veterans Affairs Medical Center, San Francisco, CA, USA. A board certified pathologist identified the cancer and normal regions on H&E stained prostate cancer tissue slides. LCM was performed by using AutoPix System (Arcturus) and Arcturus CapSure Macro LCM Caps (Applied Biosystems) following the manufacturer's instructions to isolate pure epithelial cells from normal and cancer areas of prostate tissues. Total RNA was extracted from micro-dissected FFPE tissues or cultured cells using a miRNeasy FFPE Kit (Qiagen, Valencia, CA, USA) or RNeasy mini kit (Qiagen), respectively, following the manufacturer's instructions. Total RNA was assayed for mature miRNAs using the TaqMan MicroRNA Assays in accordance with the manufacturer's instructions (Applied Biosystems). All RT reactions were run in a 7500 Fast Real Time PCR System (Applied Biosystems). Relative expression was calculated using comparative Ct. Expression of miR-466 was also validated by analyzing an independent prostate adenocarcinoma cohort publicly available at TCGA data portal now available as genomic data commons data portal (https://gdc-portal.nci.nih.gov/).

### Cell viability, migration, invasion, cell cycle and apoptosis assays

Cell viability was determined at 24, 48 and 72 h by using the CellTiter 96 AQueous One Solution Cell Proliferation Assay kit (Promega, Madison, WI, USA) according to the manufacturer's protocol. BioCoat control and Matrigel invasion chambers (BD Biosciences) and Cytoselect 24-well cell migration and invasion assay kits (Cell Biolabs, Inc., San Diego, CA, USA) were used for migration and invasion assays according to manufacturer's protocols. Fluorescence activated cell sorting (FACS) analysis was done 72 h post-transfection. The cells were harvested, washed with cold PBS, and resuspended in the nuclear stain DAPI for cell cycle analysis or stained with the 7-AAD and Annexin-V-FITC using ANNEXIN V-FITC/7-AAD KIT (BD Biosciences) for apoptosis analysis according to the manufacturer's protocol. Stained cells were immediately analyzed by FACS (BD FACSVerse; BD Biosciences).

### *In vivo* orthotopic and intracardiac mouse models

Bioluminescent PC3M-Luc-C6 cells (1 × 10^6^ cells) constitutively expressing cont-miR or miR-466 were implanted into the posterior prostatic lobe or into the left cardiac ventricle of athymic nude mice (*nu/nu*; 6–8 weeks old; Harlan Lab., IN, USA) followed by regular monitoring of tumor growth and metastatic dissemination with a live animal bioimaging system (IVIS-PerkinElmer, San Jose, CA, USA). There were ten mice in each group for orthotopic experiment, whereas 8 mice per group for intracardiac implantation. Bioluminescence signal intensity was quantified using the Living Image software (IVIS-PerkinElmer). Orthotopic experiments were performed at PerkinElmer research facility at San Jose, CA, USA.

### Antibodies and immunoblot assays

Immunoblotting was performed as described previously.^40^ In brief, protein was isolated from 70 to 80% confluent cultured cells using RIPA Extraction Reagent (Pierce Biotechnology, Rockfield, IL, USA) following the manufacturer's directions. Equal amounts of protein were resolved on 4–20% SDS polyacrylamide gels and transferred to nitrocellulose membrane. The resulting blots were blocked with 5% non-fat dry milk and probed with antibodies. All antibodies were obtained from Cell Signalling Technology Inc. Denver, MA, USA except RUNX2 and GAPDH which were purchased from Santa Cruz Biotech. Blots were visualized using Western blotting luminal reagent (sc-2048; Santa Cruz Biotechnology, Inc., Santa Cruz, CA, USA).

### Luciferase assays

Complimentary miR-466 binding sites from two different miRNA databases (microrna.org; mirdb.org) are shown in Figure 5. The RUNX2 wild-type 3′UTR and non-specific scrambled sequence 3′UTR control vectors were purchased from GeneCopoeia and named RUNX2-3′UTR vector and Control-Vector, respectively. The complete sequence of vectors is available at http://www.genecopoeia.com/. For reporter assays, cells were transiently transfected with wild-type or control reporter plasmids and miR-466 or negative miR (control). Firefly luciferase activities were measured using the Dual Luciferase Assay (Promega) 18 h after transfection and the results were normalized with Renilla luciferase. Each reporter plasmid was transfected at least three times (on different days) and each sample was assayed in triplicate.

### Statistical analysis

Statistical analyses were performed with GraphPad Prism 5 (La Jolla, CA, USA) and MedCalc version 10.3.2 (medcalc.org; Acacialaan, Ostend, Belgium). All quantified data represents an average of at least triplicate samples or as indicated. Error bars represent S.D.M. or as indicated. All tests were performed two tailed and *P-*values <0.05 were considered statistically significant. ROC were calculated to determine the potential of miR-466 to discriminate between malignant and non-malignant samples. *χ*^2^ tests were performed to determine the correlation between miR-466 expression and the clinicopathological characteristics. For recurrence-free survival analysis Kaplan–Meier curves (log-rank tests) were also performed.

## References

[bib1] Siegel RL, Miller KD, Jemal A. Cancer statistics, 2015. CA Cancer J Clin 2015; 65: 5–29. 25559415 10.3322/caac.21254

[bib2] Hughes C, Murphy A, Martin C, Sheils O, O'Leary J. Molecular pathology of prostate cancer. J Clin Pathol 2005; 58: 673–684. 15976331 10.1136/jcp.2002.003954 PMC1770715

[bib3] Komori T, Yagi H, Nomura S, Yamaguchi A, Sasaki K, Deguchi K et al. Targeted disruption of Cbfa1 results in a complete lack of bone formation owing to maturational arrest of osteoblasts. Cell 1997; 89: 755–764. 9182763 10.1016/s0092-8674(00)80258-5

[bib4] Schroeder TM, Jensen ED, Westendorf JJ. RUNX2: a master organizer of gene transcription in developing and maturing osteoblasts. Birth Defects Res C Embryo Today 2005; 75: 213–225. 16187316 10.1002/bdrc.20043

[bib5] Onodera Y, Miki Y, Suzuki T, Takagi K, Akahira J, Sakyu T et al. RUNX2 in human breast carcinoma: its potential roles in cancer progression. Cancer Sci 2010; 101: 2670–2675. 20946121 10.1111/j.1349-7006.2010.01742.x PMC11158211

[bib6] Pratap J, Lian JB, Stein GS. Metastatic bone disease: role of transcription factors and future targets. Bone 2011; 48: 30–36. 20561908 10.1016/j.bone.2010.05.035 PMC2958222

[bib7] Akech J, Wixted JJ, Bedard K, van der Deen M, Hussain S, Guise TA et al. RUNX2 association with progression of prostate cancer in patients: mechanisms mediating bone osteolysis and osteoblastic metastatic lesions. Oncogene 2010; 29: 811–821. 19915614 10.1038/onc.2009.389 PMC2820596

[bib8] Pratap J, Javed A, Languino LR, van Wijnen AJ, Stein JL, Stein GS et al. The RUNX2 osteogenic transcription factor regulates matrix metalloproteinase 9 in bone metastatic cancer cells and controls cell invasion. Mol Cell Biol 2005; 25: 8581–8591. 16166639 10.1128/MCB.25.19.8581-8591.2005 PMC1265732

[bib9] Blyth K, Cameron ER, Neil JC. The RUNX genes: gain or loss of function in cancer. Nat Rev Cancer 2005; 5: 376–387. 15864279 10.1038/nrc1607

[bib10] Stewart M, Terry A, Hu M, O'Hara M, Blyth K, Baxter E et al. Proviral insertions induce the expression of bone-specific isoforms of PEBP2alphaA (CBFA1): evidence for a new myc collaborating oncogene. Proc Natl Acad Sci USA 1997; 94: 8646–8651. 9238031 10.1073/pnas.94.16.8646 PMC23059

[bib11] Pratap J, Imbalzano KM, Underwood JM, Cohet N, Gokul K, Akech J et al. Ectopic RUNX2 expression in mammary epithelial cells disrupts formation of normal acini structure: implications for breast cancer progression. Cancer Res 2009; 69: 6807–6814. 19690135 10.1158/0008-5472.CAN-09-1471 PMC2742766

[bib12] Lim M, Zhong C, Yang S, Bell AM, Cohen MB, Roy-Burman P. RUNX2 regulates survivin expression in prostate cancer cells. Lab Invest 2010; 90: 222–233. 19949374 10.1038/labinvest.2009.128 PMC2815261

[bib13] Morrissey C, Brown LG, Pitts TE, Vessella RL, Corey E. Bone morphogenetic protein 7 is expressed in prostate cancer metastases and its effects on prostate tumor cells depend on cell phenotype and the tumor microenvironment. Neoplasia 2010; 12: 192–205. 20126477 10.1593/neo.91836 PMC2814357

[bib14] Javed A, Barnes GL, Pratap J, Antkowiak T, Gerstenfeld LC, van Wijnen AJ et al. Impaired intranuclear trafficking of RUNX2 (AML3/CBFA1) transcription factors in breast cancer cells inhibits osteolysis *in vivo*. Proc Natl Acad Sci USA 2005; 102: 1454–1459. 15665096 10.1073/pnas.0409121102 PMC547873

[bib15] Pratap J, Wixted JJ, Gaur T, Zaidi SK, Dobson J, Gokul KD et al. RUNX2 transcriptional activation of Indian Hedgehog and a downstream bone metastatic pathway in breast cancer cells. Cancer Res 2008; 68: 7795–7802. 18829534 10.1158/0008-5472.CAN-08-1078 PMC2596479

[bib16] Chua CW, Chiu YT, Yuen HF, Chan KW, Man K, Wang X et al. Suppression of androgen-independent prostate cancer cell aggressiveness by FTY720: validating RUNX2 as a potential antimetastatic drug screening platform. Clin Cancer Res 2009; 15: 4322–4335. 19509141 10.1158/1078-0432.CCR-08-3157

[bib17] Zelzer E, Glotzer DJ, Hartmann C, Thomas D, Fukai N, Soker S et al. Tissue specific regulation of VEGF expression during bone development requires Cbfa1/RUNX2. Mech Dev 2001; 106: 97–106. 11472838 10.1016/s0925-4773(01)00428-2

[bib18] Croce CM. Causes and consequences of microRNA dysregulation in cancer. Nat Rev Genet 2009; 10: 704–714. 19763153 10.1038/nrg2634 PMC3467096

[bib19] Rosenfeld N, Aharonov R, Meiri E, Rosenwald S, Spector Y, Zepeniuk M et al. MicroRNAs accurately identify cancer tissue origin. Nat Biotechnol 2008; 26: 462–469. 18362881 10.1038/nbt1392

[bib20] Garzon R, Fabbri M, Cimmino A, Calin GA, Croce CM. MicroRNA expression and function in cancer. Trends Mol Med 2006; 12: 580–587. 17071139 10.1016/j.molmed.2006.10.006

[bib21] Calin GA, Croce CM. MicroRNA-cancer connection: the beginning of a new tale. Cancer Res 2006; 66: 7390–7394. 16885332 10.1158/0008-5472.CAN-06-0800

[bib22] Calin GA, Croce CM. MicroRNA signatures in human cancers. Nat Rev Cancer 2006; 6: 857–866. 17060945 10.1038/nrc1997

[bib23] Cummins JM, Velculescu VE. Implications of micro-RNA profiling for cancer diagnosis. Oncogene 2006; 25: 6220–6227. 17028602 10.1038/sj.onc.1209914

[bib24] Volinia S, Calin GA, Liu CG, Ambs S, Cimmino A, Petrocca F et al. A microRNA expression signature of human solid tumors defines cancer gene targets. Proc Natl Acad Sci USA 2006; 103: 2257–2261. 16461460 10.1073/pnas.0510565103 PMC1413718

[bib25] Calin GA, Ferracin M, Cimmino A, Di Leva G, Shimizu M, Wojcik SE et al. A MicroRNA signature associated with prognosis and progression in chronic lymphocytic leukemia. N Engl J Med 2005; 353: 1793–1801. 16251535 10.1056/NEJMoa050995

[bib26] Bloomston M, Frankel WL, Petrocca F, Volinia S, Alder H, Hagan JP et al. MicroRNA expression patterns to differentiate pancreatic adenocarcinoma from normal pancreas and chronic pancreatitis. JAMA 2007; 297: 1901–1908. 17473300 10.1001/jama.297.17.1901

[bib27] Roldo C, Missiaglia E, Hagan JP, Falconi M, Capelli P, Bersani S et al. MicroRNA expression abnormalities in pancreatic endocrine and acinar tumors are associated with distinctive pathologic features and clinical behavior. J Clin Oncol 2006; 24: 4677–4684. 16966691 10.1200/JCO.2005.05.5194

[bib28] Yanaihara N, Caplen N, Bowman E, Seike M, Kumamoto K, Yi M et al.. Unique microRNA molecular profiles in lung cancer diagnosis and prognosis. Cancer Cell 2006; 9: 189–198. 16530703 10.1016/j.ccr.2006.01.025

[bib29] Yu SL, Chen HY, Chang GC, Chen CY, Chen HW, Singh S et al. MicroRNA signature predicts survival and relapse in lung cancer. Cancer Cell 2008; 13: 48–57. 18167339 10.1016/j.ccr.2007.12.008

[bib30] Saito M, Shiraishi K, Matsumoto K, Schetter AJ, Ogata-Kawata H, Tsuchiya N et al. A three-microRNA signature predicts responses to platinum-based doublet chemotherapy in patients with lung adenocarcinoma. Clin Cancer Res 2014; 20: 4784–4793. 25142144 10.1158/1078-0432.CCR-14-1096 PMC6329384

[bib31] Majid S, Dar AA, Saini S, Arora S, Shahryari V, Zaman MS et al. miR-23b represses proto-oncogene Src kinase and functions as methylation-silenced tumor suppressor with diagnostic and prognostic significance in prostate cancer. Cancer Res 2012; 72: 6435–6446. 23074286 10.1158/0008-5472.CAN-12-2181 PMC3940348

[bib32] Cereceda LE, Flechon A, Droz JP. Management of vertebral metastases in prostate cancer: a retrospective analysis in 119 patients. Clin Prostate Cancer 2003; 2: 34–40. 15046682 10.3816/cgc.2003.n.010

[bib33] Altieri DC, Languino LR, Lian JB, Stein JL, Leav I, van Wijnen AJ et al. Prostate cancer regulatory networks. J Cell Biochem 2009; 107: 845–852. 19492418 10.1002/jcb.22162 PMC2896787

[bib34] Yang J, Fizazi K, Peleg S, Sikes CR, Raymond AK, Jamal N et al. Prostate cancer cells induce osteoblast differentiation through a Cbfa1-dependent pathway. Cancer Res 2001; 61: 5652–5659. 11454720

[bib35] Pratap J, Lian JB, Javed A, Barnes GL, van Wijnen AJ, Stein JL et al. Regulatory roles of RUNX2 in metastatic tumor and cancer cell interactions with bone. Cancer Metastasis Rev 2006; 25: 589–600. 17165130 10.1007/s10555-006-9032-0

[bib36] Browne G, Taipaleenmaki H, Stein GS, Stein JL, Lian JB. MicroRNAs in the control of metastatic bone disease. Trends Endocrinol Metab 2014; 25: 320–327. 24811921 10.1016/j.tem.2014.03.014 PMC4075094

[bib37] Taipaleenmaki H, Browne G, Akech J, Zustin J, van Wijnen AJ, Stein JL et al. Targeting of RUNX2 by miR-135 and miR-203 impairs progression of breast cancer and metastatic bone disease. Cancer Res 2015; 75: 1433–1444. 25634212 10.1158/0008-5472.CAN-14-1026 PMC4383679

[bib38] Esquela-Kerscher A, Slack FJ. Oncomirs - microRNAs with a role in cancer. Nat Rev Cancer 2006; 6: 259–269. 16557279 10.1038/nrc1840

[bib39] Ell B, Mercatali L, Ibrahim T, Campbell N, Schwarzenbach H, Pantel K et al. Tumor-induced osteoclast miRNA changes as regulators and biomarkers of osteolytic bone metastasis. Cancer Cell 2013; 24: 542–556. 24135284 10.1016/j.ccr.2013.09.008 PMC3832956

[bib40] Majid S, Dar AA, Saini S, Yamamura S, Hirata H, Tanaka Y et al. MicroRNA-205-directed transcriptional activation of tumor suppressor genes in prostate cancer. Cancer 2010; 116: 5637–5649. 20737563 10.1002/cncr.25488 PMC3940365

