# Supplementary material for: MicroRNA-466 inhibits tumor growth and bone metastasis in prostate cancer by direct regulation of osteogenic transcription factor RUNX2
Source: Cell Death Dis. 2017 Jan 26;8(1):e2572–. doi: 10.1038/cddis.2017.15 (PMC5386393; doi:10.1038/cddis.2017.15)
Supplement: Supplementary Information [file cddis201715x1.doc]

**Supplemental Table 1. Clinicopathologic characteristics of VAMC plus TCGA data cohorts of prostate cancer patients.**

**Characteristics Patient set†**

**Age, Years Number of patients**

Mean 62

Median 68

Range 48-81

**T-stage (n=90)**

pT2 58

pT3-pT4 26

**Gleason Score (n=92)**

<7 41

7 33

>7 9

**PSA failure (n=34)**

Yes 34

**Pathological diagnosis**

Adenocarcinoma

**†**miR-466 expression in VAMC cohort was determined from matched laser captured microdisected tissues.
